# A GREEN ACTIVITY PRESCRIPTION PROGRAM FOR CHINESE AMERICANS LIVING WITH DEMENTIA

**DOI:** 10.1093/geroni/igad104.2342

**Published:** 2023-12-21

**Authors:** Hayley Belli, Rebecca Lassell, Jun Luo, Lorna Thorpe, Jennifer Wong, Stella Yi, Abraham Brody

**Affiliations:** New York University, New York City, New York, United States; New York University, New York City, New York, United States; New York University, New York City, New York, United States; New York University, New York City, New York, United States; New York University School of Medicine, New York City, New York, United States; New York University School of Medicine, New York City, New York, United States; NYU Rory Meyers College of Nursing, New York City, New York, United States

## Abstract

Asian Americans account for approximately 161,000 cases of Alzheimer’s disease and related dementias (AD/ADRD) among adults ages 65 and older in the United States. Chinese Americans have among the highest incidences of AD/ADRD, and have experienced underdiagnosis, cultural stigmas, and less access to dementia care. In New York City specifically, 28.6% of Chinese American seniors live in poverty, leading to environmental racism, a lower likelihood of engagement in activities, decreased quality of life, and an increased risk of cognitive decline and developing dementia. Recommendations to support activity engagement among Chinese Americans include fostering social cohesion and linking individuals to local activity resources. Green activity prescriptions (GAPs) are one approach to achieving these objectives, and combine nature, social contexts, and meaningful activities to improve health and well-being. GAPs are co-designed with participants based on their interests, needs, and available community resources. Examples include: animal-assisted interventions, gardening, green exercise (walking), and conservation projects (composting). Past studies have provided evidence that these components have improved well-being for PLWD, supported function, and provided meaningful engagement. Yet, these early studies neglected Chinese Americans. GAPs have potential to support the health and well-being of Chinese American PLWD by addressing health inequalities and providing accessible, low-cost care, all while sustaining local green resources. Here we present the study design and implementation outcomes, including recruitment and engagement strategies, of a GAP intervention utilizing a community-based participatory approach aimed at supporting the health and well-being of Chinese American PLWD residing in lower-socioeconomic households within Brooklyn, NY.

